# Noncommunicable Disease Risk Factors and Mobile Phones: A Proposed Research Agenda

**DOI:** 10.2196/jmir.7246

**Published:** 2017-05-05

**Authors:** Adnan A Hyder, Adaeze C Wosu, Dustin G Gibson, Alain B Labrique, Joseph Ali, George W Pariyo

**Affiliations:** ^1^ Johns Hopkins Bloomberg School of Public Health Department of International Health Baltimore, MD United States; ^2^ Berman Institute of Bioethics Johns Hopkins University Baltimore, MD United States; ^3^ Johns Hopkins Bloomberg School of Public Health Department of Epidemiology Baltimore, MD United States

**Keywords:** mHealth, noncommunicable disease, mobile phone, research agenda, survey

## Abstract

Noncommunicable diseases (NCDs) account for two-thirds of all deaths globally, with 75% of these occurring in low- and middle-income countries (LMICs). Many LMICs seek cost-effective methods to obtain timely and quality NCD risk factor data that could inform resource allocation, policy development, and assist evaluation of NCD trends over time. Over the last decade, there has been a proliferation of mobile phone ownership and access in LMICs, which, if properly harnessed, has great potential to support risk factor data collection. As a supplement to traditional face-to-face surveys, the ubiquity of phone ownership has made large proportions of most populations reachable through cellular networks. However, critical gaps remain in understanding the ways by which mobile phone surveys (MPS) could aid in collection of NCD data in LMICs. Specifically, limited information exists on the optimization of these surveys with regard to incentives and structure, comparative effectiveness of different MPS modalities, and key ethical, legal, and societal issues (ELSI) in the development, conduct, and analysis of these surveys in LMIC settings. We propose a research agenda that could address important knowledge gaps in optimizing MPS for the collection of NCD risk factor data in LMICs and provide an example of a multicountry project where elements of that agenda aim to be integrated over the next two years.

## Background

The World Health Organization (WHO) estimates that 67% of the 56 million deaths that took place globally in 2012 were due to noncommunicable diseases (NCDs), in particular, cardiovascular diseases (42.6%), cancers (21.7%), chronic respiratory diseases (10.7%), and diabetes (4.0%) [1]. About 75% of all NCD deaths took place in low- and middle-income countries (LMICs) where nearly half of all deaths occurred in persons aged under 70 years [1]. The WHO projects a rise in the number of NCD deaths from 36 million in 2008 to 55 million by 2030 if effective steps are not taken to curb the epidemic [2]. Furthermore, deaths from NCDs for people younger than 70 years old is projected to increase from 10.8 million in 2010 to 15.4 million in 2050 [3]. Four key risk factors responsible for a majority of NCDs are tobacco use, unhealthy diet, sedentary lifestyle, and excessive use of alcohol—all behavioral and largely modifiable [4]. These factors, as well as loss of employment due to NCD-related disability, combined with the long duration and complexity of NCD treatment pose additional challenges to poverty reduction and sustainable development [5].

Key to global efforts to prevent and control NCDs is national surveillance. At the national level, surveillance has three primary components: exposures (behavioral, physiologic, metabolic, social determinants), outcomes (NCD-specific morbidity and mortality), and health system capacity and response (infrastructure, policies, plans, access to health care, and partnerships) [4]. The WHO STEP-wise approach to NCD risk factor surveillance (STEPS) provides a standardized framework and toolkit for monitoring national levels of major NCD risk factors (exposures) [6]. STEPS consists of three levels of data collection: questionnaires, physical measurements, and biochemical measurements [6]. Similar to other household surveys, the STEPS questionnaires are conducted through face-to-face interviews, thus requiring significant time and resource commitments.

### Targets and Indicators

In 2013, the WHO developed an NCD global action plan and monitoring framework, establishing 9 voluntary targets and 25 indicators that have to be achieved to attain a 25% reduction in premature mortality from the four major NCDs (ie, cardiovascular diseases, cancers, chronic respiratory diseases, and diabetes) by 2025 [7]. A summary of these targets and indicators is provided in Table 1. Targets include a 25% relative reduction in the risk of premature death from the four major NCDs, a 30% relative reduction in the prevalence of current tobacco use, and a 30% relative reduction in mean population intake of salt or sodium [1,2,7]. The WHO NCD global action plan also calls for surveillance efforts to incorporate cross-sectorial engagement [1]. Despite substantial progress over the past decade, reliable, high-quality data on the prevalence, incidence, and consequences of NCDs remain less available in some countries [1].

One promising approach increasingly being explored for public health surveillance involves mobile phones. An emergent field, mobile health (mHealth), describes medical and public health activities that leverage the global proliferation of cellular networks and mobile phone ownership (or access) to improve population and clinical health outcomes. Device functionalities such as voice calls and short message services (SMS)—often referred to as text messages—global positioning system (GPS), as well as other wearable and connected devices may be harnessed to provide data and insight on individuals and populations [8,9]. Currently, there are over 7.4 billion wireless subscriptions globally, with the majority (78%) in LMICs [10]. The UN International Telecommunications Union estimates 2016 global phone ownership at 99.7 mobile phone subscriptions per 100 persons, a significant increase compared with 41.7 mobile phone subscriptions per 100 persons a decade ago [10,11]. It is this ubiquitous connectivity to cellular networks that potentially makes large proportions of a population accessible through their mobile phones.

In view of the increasing disease burden, the intersecting need for NCD data in LMICs and the near-universal population access to mobile phones in a growing number of countries presents an opportunity for public health. As cellular technologies leapfrog the need for traditional landline infrastructure, public health programs could conduct remote, population-level surveys by reaching out to community members through their own cell phones. Can such a widely dispersed technology have the potential to assist in the achievement of the WHO NCD global action plan targets? How can mobile phone surveys (MPS) be used for population-level NCD risk factor surveillance? What critical technology, sampling, and methodological questions need to be addressed before leveraging this potential? In this paper, we present the potential for MPS to collect such data, review key research issues, and introduce a multicountry effort that seeks to partly respond to this public health challenge. We draw on a selected review of literature, key global reports, and experience of the authors to propose a research agenda and report on efforts underway to address it. It is believed that this paper will facilitate a global dialog and action to enhance the use of MPS for NCD, and potentially other public health risk factor surveillance.

**Table 1:** Summary of the World Health Organization (WHO) 9 voluntary targets and 25 indicators for global noncommunicable disease (NCD) monitoring [1].

Element		Targets	Relevant indicators
**Morbidity and mortality**			
	Premature mortality from NCD	25% reduction in premature mortality from NCD	Unconditional probability of death between ages 30 and 70 years from the 4 main NCDs
			Cancer incidence, by type of cancer, per 100,000 population
**Risk Factors**			
*Behavioral risk factors*			
	Harmful use of alcohol	10% reduction in harmful use of alcohol	Total (recorded, unrecorded) alcohol per capita (in ≥15 years old) consumption within a calendar year in liters pure alcohol
			Age-standardized prevalence of heavy episodic drinking among adolescents and adults
			Alcohol-related morbidity and mortality among adolescents and adults
	Physical inactivity	10% reduction in prevalence of physical inactivity	Prevalence of insufficiently active adolescents, defined as <60 min of moderate or vigorous intensity activity daily
			Age-standardized prevalence of insufficiently active persons, aged ≥18 years (defined as <150 min moderate activity per week, or equivalent)
	Salt or sodium intake	30% reduction in mean population intake of salt or sodium	Age-standardized mean population intake of salt (sodium chloride) per day in grams in persons ≥18 years old
	Tobacco use	30% reduction in prevalence of tobacco use	Prevalence of current tobacco use among adolescents
			Age-standardized prevalence of current tobacco use among persons ≥18 years old
*Biological risk factors*			
	Raised blood pressure	25% reduction in prevalence of raised blood pressure or contain the prevalence of raised blood pressure	Age-standardized prevalence of raised blood pressure among persons ≥18 years old (defined as systolic blood pressure ≥140 mmHg and/or diastolic blood pressure ≥90 mmHg); and mean systolic blood pressure
	Diabetes and obesity	0% increase in diabetes or obesity	Age-standardized prevalence raised blood glucose or diabetes among ≥18 years old (fasting plasma glucose ≥7.0 mmol/L (126 mg/dL) or on medication for raised blood glucose)
			Prevalence overweight or obesity in adolescents (WHO growth reference for school-aged children and adolescents)
			Age-standardized prevalence of overweight or obesity ≥18 years (body mass index ≥25 kg/m²; overweight ≥30 kg/m² obesity)
*Additional indicators*			Age-standardized mean proportion of total energy intake from saturated fatty acids in persons ≥18 years old
			Age-standardized prevalence (≥18 years old) consuming less than five total servings (400 g) of fruit or vegetables per day
			Age-standardized prevalence raised total cholesterol aged ≥18 years (total cholesterol ≥5.0 mmol/L or 190 mg/dL)
**National Response Systems**			
	Drug therapies to prevent heart attacks and strokes	≥50% coverage in eligible individuals in drug therapy and counseling	Proportion of eligible persons (defined as ≥40 years old with a 10-year cardiovascular risk of ≥30%, including those with existing CVD) receiving drug therapy and counseling (including glycemic control) to prevent heart attacks and strokes
	Essential NCD medicines and basic technologies to treat major NCDs	80% coverage for technologies and essential NCD medicines to treat NCD in public and private facilities	Availability and affordability of quality, safe and efficacious essential NCD medicines, including generics, and basic technologies in both public and private facilities
*Additional indicators*			Access to palliative care assessed by morphine-equivalent consumption of strong opioid per death from cancer
			Adoption of national policies that limit saturated fatty acids and virtually eliminate partially hydrogenated vegetable oils in the food supply
			Availability of cost-effective and affordable vaccines against human papillomavirus
			Policies to reduce the impact on children of marketing of foods and nonalcoholic beverages high in saturated fats, trans fatty acids, free sugars, or salt
			Vaccination coverage hepatitis B by number of third doses of Hepatitis-B vaccine (HepB3) administered to infants
			Proportion of women 30-49 years screened for cervical cancer at least once

^a^NCD: noncommunicable disease.

^b^mmol: millimole.

^c^mg: milligram.

^d^dL: deciliter.

^e^WHO: World Health Organization.

^f^CVD: cardiovascular disease.

### Potential of Mobile Phone Surveys for NCD Data

Access to mobile phones has increased globally, reaching more than 100% saturation in some LMICs [10,11]. For the period 2010-2015, for example, mobile phone subscriptions per 100 people increased 30-85% in the following countries: Bangladesh (45 to 83), Ghana (85 to 130), Lebanon (66 to 87), Papua New Guinea (28 to 47), Uganda (38 to 50), and Zambia (41 to 74) [11]. mHealth technology is already being incorporated into public health data collection due to its potential to save time and costs, as well as its reliability across both highly developed urban centers and remote, rural areas. A recent review described 12 common mHealth strategies, from surveillance and training to point-of-care diagnostics, registries or vital events tracking, as well as commodities management [12]. Some advantages of mHealth-empowered surveillance include instantaneous collection and sharing of data (across large samples), enabling analysis of results by demographic and spatial characteristics, significant savings in the amount of time needed to obtain data from remote geographic areas (without the need to go there like traditional household surveys), and potential cost savings from reductions in human resources needed to administer questionnaires and enter data [12].

It was found that 83% of the 112 WHO member states responding to a 2014 survey reported having one or more mHealth initiatives; the most frequently reported categories were call centers or helplines, emergency toll free telephone services, and mobile telemedicine (reported by 49-59% of countries). Least frequent uses of mHealth were health surveys, surveillance, awareness, and decision support (reported by 19-26% of countries) [8]. The WHO noted that these findings appeared to be incongruous with peer-reviewed literature, which tends to report more frequent use of mHealth for data collection and disease surveillance. Possibly, those responding to the survey may not have been aware of mHealth programs in the nongovernmental or private sectors, or it is possible that nongovernmental organizations (NGO) or government programs are slow to share activities and findings in the academic literature [13,14]. To promote better integration, scale-up, and sustainability of mHealth initiatives, the mHealth Assessment and Planning for Scale (MAPS) toolkit provides detailed self-assessment for project teams to measure the scalability of their mHealth products, as well as planning and guidance strategies for surmounting barriers to scale [15].

Limited evidence exists on the comparative effectiveness of MPS modalities in LMICs though a variety of options are available. Three frequently employed modalities are interactive voice response (IVR), SMS, and computer-assisted telephone interview (CATI). IVR involves prerecorded audio messages or instructions given over the phone by a computer application; CATI involves one person interviewing another via the phone; and SMS involves communication between devices using short text messages [16]. Although studied in high-income countries, there is limited empirical evidence on the factors that influence MPS response, completion, and attrition, as well as ways to maximize these key metrics in LMIC settings across any of these modalities [17,18].

In one comparative study, 630 Lebanese adults selected through multistage stratified cluster sampling were respondents for a face-to-face (40-min) survey adapted from the WHO STEPS, and a mobile phone interview (abridged version of NCD questions). The study reported high percent agreements (89.5-95.6%) and kappa statistics (κ=.79 to .91) for past-year alcohol consumption, ever smoking, and current cigarette smoking; and over 90% agreement for diagnoses of hypertension, diabetes, hyperlipidemia, and heart disease (κ=.87 for diabetes; κ=.66 to .68 for hypertension, hyperlipidemia, and heart disease). Mobile phone interviews also saved about US $14 per person interviewed, when compared with face-to-face methods [19]. The World Bank compared MPS modalities in Peru and Honduras to collect frequent and nationally representative data by sending out 677 invitations on SMS, 383 for IVR, and 384 for CATI. Attrition rates were high for IVR (75-81%) and SMS (70-79%) compared with CATI (49-61%); and modalities had different cost per interview including face-to-face (US $40), CATI (US $25), IVR (US $17), and SMS (US $8). In addition, answers to SMS and IVR were significantly different from face-to-face answers, with CATI responses being almost the same as face-to-face responses [16].

The evidence base around the efficacy and impact of mHealth programs is growing rapidly, with increasing numbers of projects being evaluated with methodological rigor [20,21]. However, a number of issues related to the optimization and conduct of MPS have been highlighted in the literature and appear to be largely unaddressed. These include concerns around equity, the optimal timing of surveys, appropriate financial incentives, and the quality and accuracy of responses [22,23]. As a nascent field, many mHealth strategies are still being tested and have not all been carefully evaluated; for example, a 2012 review of SMS interventions for NCD prevention in LMICs found that only 5 of 34 projects included in the review provided an evaluation of impact [22].

The proliferation of mobile phones, and the critical gaps raised by WHO and the literature highlight both the potential for MPS and the need for rigorous investigations of the feasibility, validity, and comparative effectiveness of different MPS modalities. In addition, there is a need to understand the ethical, legal, and societal issues (ELSI) around use of mobile phones for collecting NCD risk factors. These issues give rise to a rich research and development agenda proposed below.

## NCD Risk Factors and Mobile Phone Survey: A Research Agenda

We propose a research and development agenda for NCD risk factors and MPS. The eventual purpose of the proposed agenda is to help standardize operating procedures for MPS, which will allow for comparisons of NCD risk factors within and across sites, and over time. Within this overall purpose, we propose specific goals. First, to answer key questions on how to design and deliver robust MPS to collect NCD risk factors; second, to assess the comparative effectiveness and costs of MPS modalities (eg, IVR, CATI, SMS); and third, to determine key ELSI in the development, in conduct, analysis, and reporting of MPS in LMICs. Each is further described below with proposed research objectives. These goals and specific objectives are also summarized in Table 2.

Under the *first goal* (to answer key questions on how to design and deliver robust MPS on NCDs), one research objective is to assess the usability of different ways of delivering MPS (eg, voice calls, recorded voice surveys—commonly referred to as interactive voice response (IVR)—or by text message) and community perceptions and willingness to complete MPS. This will probably require qualitative methods such as key informant interviews, focus group discussions, and user-group testing. Participants’ perceptions and willingness to complete and ability to navigate MPS delivery modalities such as IVR, SMS, and CATI can be assessed in order to provide a user-designed MPS [24]. These formative efforts can help inform translation and wording of questions, and suggest ways to improve MPS response rates. Results can be used to improve MPS platform design and questionnaires.

A second objective is to assess the role of different incentive amounts in MPS. Quasi-experimental or randomized controlled trial designs can be used to assess the effect of incentives on standardized contact, response, completion, and refusal rates in a MPS [25]. Studies will need to assess how different monetary incentive amounts; timing of incentive delivery (eg, beginning of MPS vs end); and nature of incentive structure (eg, fixed amount vs lottery) affect MPS metrics. These studies will need specific sample size calculations and calculation of key survey metrics most likely stratified by key demographic characteristics. Important issues around sampling, weighting, and stratification also remain understudied for MPS under conditions of varying mobile phone ownership and/or access [26].

A third objective will be to assess the impact of MPS introductory messages. This will, for example, necessitate studies that assess the effect of MPS introductory message on contact, response, completion, refusal, and attrition rates. Research could further examine the introduction content (motivational vs informational), voice type (male vs female), and the introduction’s character (casual voice vs formal) on key survey metrics by demographic characteristics. Exploring different sampling frames of mobile phone numbers is a fourth objective to assess the effect of the sampling frame on representativeness, response rate, completion rate, and costs of MPS. For example, the benefit of using random digit dialing (RDD) compared with a list of preexisting phone numbers, for example, from mobile network operators or previous surveys has not been characterized. Finally, the impact of NCD-specific questions (in modules) on survey attrition needs to be studied. NCD modules in existing surveys are groups of common-themed risk factor questions, such as diet and alcohol, and can be delivered in different order (or randomized) to assess survey impact by question number and content (also tests cultural sensitivities) by key demographics. The research agenda under this first goal will help optimize the delivery of MPS.

Under the *second goal* (to assess the comparative effectiveness of MPS modalities), it is important to understand the role and impact of different channels of communication with phone owners. One objective is to assess the effect of MPS modalities (eg, CATI vs IVR) on key metrics and performance indicators, for example, by administering the same survey content to a list of participants using two or more modalities. The costs of modality setup, deployment, and maintenance also need to be assessed to perform intermodal cost-comparisons. A second objective is to compare national or subnational estimates of NCD risk factor indicators between MPS modalities—this could be done by comparing NCD risk factor distributions captured using different MPS modalities. Variability in response to different questions, modules and risk factor prevalence captured by multiple modalities can be ascertained.

A third objective is to document MPS intermodal reliability and response consistency for NCD risk factors. This could be done by allocating (or randomizing) participants into two or more arms (eg, IVR and CATI) to establish congruency in responses for the same questions. Participants in one arm could receive one modality followed by another modality a few days later. Crossover designs might also be possible for an assessment of response consistency, adjusted for the risk of priming after exposure to the prior modality. Differences in the reported prevalence of NCD risk factors can also be evaluated to quantify possible incongruences in responses between MPS modalities. These objectives under the second research goal will answer questions on comparative effectiveness assessment in MPS in LMIC settings, as well as questions as to which modality provides better information on key survey metrics.

Under the *third goal* (determine key ELSI) relevant issues need to be explored in the development, conduct, analysis, and reporting of MPS in LMICs. Objectives under this goal are to (1) determine key ethical issues encountered in the conduct of MPS; (2) identify common and preferred practices for obtaining individual consent or permission for MPS to ensure voluntary, informed data collection; (3) document commonly encountered regulatory complexities and challenges that arise when conducting MPS and how they have been addressed; and (4) identify key societal goals and values that are supportive of MPS and how these are balanced against other important interests (eg, expectations of privacy and confidentiality).

A systematic review of the literature is needed to collect the most common ELSI and collate them according to broad themes. Data collection with researchers, public health practitioners, programmers, policymakers, and other stakeholders of MPS may help define and assess the key ELSI, how they have been addressed in practice, and how well they are reflected in existing reports and theoretical frameworks identified by global stakeholders. Additional conceptual work may be required in this space to understand which ethics principles and values are being satisfied and which may be tested using MPS. Equally important is the critical examination of factors that influence whether, and to what extent, various ELSI apply across different types of mHealth and digital health activities. This research goal will provide crucial perspectives and data to inform the responsible implementation of MPS in, by and for LMICs. The three research goals and possible objectives described above comprise an initial proposed research agenda for MPS and NCD risk factor data collection with a special focus on LMICs (Table 2).

## Integration Into Data Projects: An Opportunity

Implementing the proposed research agenda will require both resources and creativity, either as standalone activities or integrated into other agendas. As such, we introduce in this section, an opportunity for integrating this agenda into a stream of work around data for health and NCD. The Bloomberg Data for Health Initiative (BD4HI) is supported by Bloomberg Philanthropies and the Australian Government’s InnovationXchange to improve the health of populations through strengthening public health data [27]. It involves multiple partners working in several sites including at national and subnational levels (Table 3). The project has an arm focused on strengthening vital statistics including birth and death registration in several countries. It also has an NCD arm implementing both nationally representative WHO STEPS survey and MPS using different mHealth platforms to generate comparable data across countries. The MPS are being designed to use or closely approximate the standardized STEPS survey with questions on demographics, tobacco use, alcohol consumption, diet, physical activity, and history of blood pressure and diabetes diagnoses and medications [6]. Finally, the project also has an arm focused on strengthening data impact and use of data for policies in countries.

It is in the context of this project that we proposed to integrate an initial research agenda for MPS (Table 2). Key to success of our research agenda is an understanding that this research would seek to inform MPS activities at the national level by feeding results to implementing partners and governments through our in-country collaborating institutions. In order to make this a realistic program of work, we decided to (1) initially focus on a few (2-4) selected countries in either the African, Middle Eastern, or Asian regions; (2) select two MPS modalities only for testing—IVR and CATI for now; and (3) work only at the subnational level. These initial research efforts will help inform future national level rollout in other countries considering adopting use of MPS. Table 2 includes a summary of our project-specific research agenda based on these decisions; this agenda incorporates elements of each of the three goals described above.

**Table 2:** Proposed research agenda for mobile phone survey (MPS) and noncommunicable disease (NCD) risk factor data collection

Goals	Objectives	BD4HI
Goal 1: Answer key questions on how to design and deliver robust MPS on NCDs.	Exploring the usability or technical requirements of MPS modalities, community perceptions, and willingness to take MPS.	The project will (1) identify lessons learned and challenges from individuals with experience of MPS (via key informant interviews); (2) understand community acceptance and willingness to respond to IVR (via focus group discussions); and (3) examine and refine the usability of an NCD risk factor survey delivered through an IVR platform (via semi-structured interviews).
	Exploring the impact of incentive amounts, incentive timing, and incentive structure on key MPS metrics.	Test impact of different incentive factors on key survey metrics; participants to receive different incentive (1) amounts (including none), (2) timing (prepaid to poststudy), and (3) structures (fixed, lottery).
	Exploring the impact of the content, type, and modality of introductory messages on key MPS metrics.	Exploring the impact of different content (informational, motivational) and voice (male, female) of IVR introduction.
	Exploring the impact of different sampling frames on key MPS metrics.	Assess the benefit of using random digit dialing (RDD) compared with a list of preexisting phone numbers
	Exploring the impact of specific questions and their order on key MPS metrics.	Examine impact of different orders of NCD modules (eg, diet, tobacco use, alcohol) on key survey metrics.
Goal 2: Assess the comparative effectiveness of MPS modalities	Assess impact of MPS modality on key metrics, performance characteristics, and costs of MPS.	Participants will respond to either CATI or IVR using the same questionnaire; and response characteristics will be studied.
	Compare national or subnational indicators between MPS modalities.	Responses of participants to IVR and CATI surveys will be compared.
	MPS intermodal reliability and response consistency.	Participants will be randomized to one of two arms: IVR then CATI, or CATI then IVR. The questionnaires used in both study arms will be the same. Crossover design will allow for an assessment of response consistency
Goal 3: Explore ethical, legal and societal issues (ELSI) in the development, conduct, analysis, and reporting of MPS	Determine key ethical issues in the conduct of MPS.	Conduct of a systematic review of the literature to collect the most common ELSI and collate them according to broad themes.
	Describe common and preferred practices for obtaining individual consent or permission for MPS.	Conduct a survey of researchers, programmers, users, and stakeholders of MPS on and from LMICs to help define the prevalence of key ELSI; and how they have been addressed.
	Document commonly encountered regulatory complexities when conducting MPS and how have they been addressed.	
	Identify key societal goals and values that are supportive of MPS and how these are balanced against other important interests.	

^a^BD4HI: Bloomberg Data for Health Initiative.

^b^MPS: mobile phone surveys.

^c^NCDs: noncommunicable diseases.

^d^IVR: interactive voice response.

^e^CATI: computer-assisted telephone interview.

^f^ELSI: ethical, legal, and societal issues.

^g^LMICs: low- and middle-income countries.

**Table 3:** Bloomberg Data for Health Project—key goals, components, and partners.

Key features	Description
Overall goal	To improve the health of populations through strengthening collection and use of public health data
Components	Civil Registration and Vital Statistics (CRVS) Improvement Noncommunicable Disease Risk Factor Surveillance Strategic Use of Data for Policy and Planning
Partners	Centers for Disease Control and Prevention, USA Johns Hopkins Bloomberg School of Public Health, USA University of Melbourne, Australia Vital Strategies World Health Organization
Donors	Bloomberg Philanthropies Australian Government

## Discussion

Currently, there are over 7 billion mobile phone subscriptions around the globe [10]. The majority of subscriptions are in LMICs, which are undergoing demographic and epidemiologic transitions where NCDs are catching up to infectious diseases as leading causes of death. To address this challenge, countries need cost-effective, timely, and systematic means of conducting surveillance and tracking progress toward globally agreed targets for NCD prevention and control [6,28]. Mobile phones present an opportunity to conduct rapid risk factor surveillance that can be used to inform resource allocation, as well as evaluate NCD prevention and control efforts and policies [29]. Addressing unresolved questions about maximizing key metrics for MPS, comparative effectiveness of MPS, and ELSI in the development, conduct, analysis, and reporting of MPS, would aid collection of timely and quality information about NCD risk factors [17,18]. The eventual goal of the proposed research agenda is to help standardize operating procedures for MPS, which will allow for comparisons of NCD risk factors within and across sites, and over time.

Despite the many advantages they present, it is important to note that NCD risk factor MPS probably would not obviate the need for in-person data collection. Physical measurements such as blood pressure, weight or height, and waist circumference, or obtaining blood samples for analyses (eg, for lipids, glucose), need to be conducted in-person. The MPS can complement an NCD surveillance system that includes a STEPS or similar survey done less frequently, which together can inform prevention and policy decisions [30]. The surveys, of course, have to be coupled with other aspects of the WHO surveillance framework including outcomes (ie, disease rates, deaths), health system capacity, and response (ie, infrastructure, policies or plans, access to care or medicines, partnerships) [4].

The BD4HI offers a unique opportunity to test MPS technology and delivery options in real world settings, while enhancing our understanding of the norms and values that are likely to influence global implementation. Proof of concept on the use of this technology for NCD risk factor data collection could help countries to complement existing mechanisms for monitoring progress in their interventions toward achieving commitments for NCD global targets. We welcome the global NCD and mHealth communities to provide further input on the proposed research agenda and hope it will inform research projects in the coming years.

## Abbreviations

BD4HI: Bloomberg Data for Health Initiative
CATI: computer-assisted telephone interview
CRVS: Civil Registration and Vital Statistics
CVD: cardiovascular disease
ELSI: ethical, legal, and societal issues
GPS: global positioning system
IVR: interactive voice response
LMIC: low- and middle-income countries
MAPS: mHealth Assessment and Planning for Scale
MPS: mobile phone surveys
NCD: noncommunicable disease
NGO: nongovernmental organization
RDD: random digit dialing
SMS: short message service
STEPS: WHO STEP-wise approach to NCD risk factor surveillance
WHO: World Health Organization
